# Replication Kinetics, Cell Tropism, and Associated Immune Responses in SARS-CoV-2- and H5N1 Virus-Infected Human Induced Pluripotent Stem Cell-Derived Neural Models

**DOI:** 10.1128/mSphere.00270-21

**Published:** 2021-06-23

**Authors:** Lisa Bauer, Bas Lendemeijer, Lonneke Leijten, Carmen W. E. Embregts, Barry Rockx, Steven A. Kushner, Femke M. S. de Vrij, Debby van Riel

**Affiliations:** a Department of Viroscience, Erasmus Medical Center, Rotterdam, The Netherlands; b Department of Psychiatry, Erasmus Medical Center, Rotterdam, The Netherlands; University of Pittsburgh School of Medicine

**Keywords:** neurotropism, hiPSC neurons, coronavirus, SARS-CoV-2, COVID-19, influenza A virus, H5N1 virus, IL-8, interferon, influenza virus

## Abstract

Severe acute respiratory syndrome coronavirus 2 (SARS-CoV-2) infection is associated with a wide variety of neurological complications. Even though SARS-CoV-2 is rarely detected in the central nervous system (CNS) or cerebrospinal fluid, evidence is accumulating that SARS-CoV-2 might enter the CNS via the olfactory nerve. However, what happens after SARS-CoV-2 enters the CNS is poorly understood. Therefore, we investigated the replication kinetics, cell tropism, and associated immune responses of SARS-CoV-2 infection in different types of neural cultures derived from human induced pluripotent stem cells (hiPSCs). SARS-CoV-2 was compared to the neurotropic and highly pathogenic H5N1 influenza A virus. SARS-CoV-2 infected a minority of individual mature neurons, without subsequent virus replication and spread, despite angiotensin-converting enzyme 2 (ACE2), transmembrane protease serine 2 (TMPRSS2), and neuropilin-1 (NPR1) expression in all cultures. However, this sparse infection did result in the production of type III interferons and interleukin-8 (IL-8). In contrast, H5N1 virus replicated and spread very efficiently in all cell types in all cultures. Taken together, our findings support the hypothesis that neurological complications might result from local immune responses triggered by virus invasion, rather than abundant SARS-CoV-2 replication in the CNS.

**IMPORTANCE** Infections with the recently emerged severe acute respiratory syndrome coronavirus 2 (SARS-CoV-2) are often associated with neurological complications. Evidence suggests that SARS-CoV-2 enters the brain via the olfactory nerve; however, SARS-CoV-2 is only rarely detected in the central nervous system of COVID-19 patients. Here, we show that SARS-CoV-2 is able to infect neurons of human iPSC neural cultures but that this infection is abortive and does not result in virus spread to other cells. However, infection of neural cultures did result in the production of type III interferon and IL-8. This study suggests that SARS-CoV-2 might enter the CNS and infect individual neurons, triggering local immune responses that could contribute to the pathogenesis of SARS-CoV-2-associated CNS disease.

## INTRODUCTION

Neurological manifestations are present in a substantial proportion of patients suffering from the respiratory coronavirus disease 2019 (COVID-19). Symptoms comprise loss of smell (anosmia), loss of taste (hypogeusia), headache, fatigue, nausea, and vomiting (1–3). Additionally, more severe neurological complications such as seizures, confusion, cerebrovascular injury, stroke, encephalitis, encephalopathies, and altered mental status are being increasingly reported in hospitalized patients (4–6).

It remains to be established whether the reported neurological manifestations are a direct consequence of local invasion by severe acute respiratory syndrome coronavirus 2 (SARS-CoV-2) into the central nervous system (CNS), an indirect consequence of the associated systemic immune responses, or a combination of the two. In human and animal models, it has been shown that SARS-CoV-2 is able to replicate in the olfactory mucosa (7, 8), suggesting that the olfactory nerve could function as an important route of entry into the CNS (9), as observed previously for other respiratory viruses (10). Postmortem brain tissue analyses of fatal COVID-19 cases have revealed mild neuropathological changes which might be related to hypoxia, as well as pronounced neuroinflammation in different regions of the brain (11). In the majority of cases, neither SARS-CoV-2 viral RNA, nor virus antigen, could be detected in the CNS (5, 12). In line with this, SARS-CoV-2 viral RNA has rarely been detected in the cerebrospinal fluid (CSF) of COVID-19 patients with neurological symptoms (13–15). Together, this suggests that SARS-CoV-2 might enter the CNS but be unable to replicate there efficiently.

**FIG 1:**
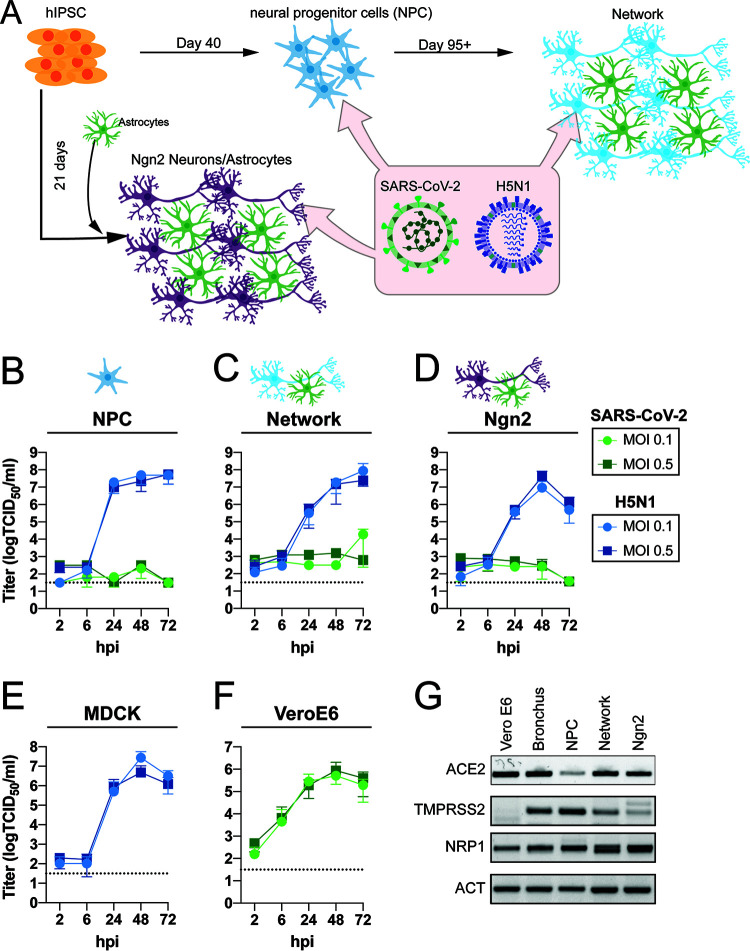
SARS-CoV-2 does not replicate in hiPSC-derived neural (co-)cultures in contrast to H5N1 virus. (A) A schematic depiction of the different hiPSC-derived differentiation strategies of the neural cultures. hiPSCs are differentiated into neural progenitor cells (NPCs) and subsequently into mixed neural network cultures containing mixed neurons and astrocytes. Alternatively, hiPSCs are differentiated into excitatory neurons by inducing overexpression of Ngn2; these are grown in a coculture with hiPSC-derived astrocytes. (B to D) Growth kinetics of SARS-CoV-2 or H5N1 virus, in hiPSC-derived NPCs (B), mature neural networks (C), or Ngn2 cocultures (D) using an MOI of 0.1 and 0.5. (E and F) As positive controls, MDCK (E) and VeroE6 (F) cells were infected with H5N1 virus or SARS-CoV-2, respectively. Data represent mean ± standard deviation (SD) from three independent experiments. Every growth curve was performed in either biological duplicates or triplicates. (G) Presence of the host factors angiotensin-converting enzyme 2 (ACE2), transmembrane protease serine 2 (TMPRSS2), and neuropilin-1 (NRP1) of the neural cultures was determined with PCR. As controls for the expression of ACE2 and TMPRSS2, bronchus-bronchiole organoids were used. The uncropped agarose gels are displayed in Fig. S2.

In the brain, viruses encounter a variety of different cell types such as neurons, astrocytes, and microglia. Investigations of CNS cell-type-specific infection of SARS-CoV-2 have been inconsistent (16–23). Most studies have investigated virus replication by the detection of viral RNA but have not reported whether infectious progeny viruses are produced during the course of infection. In order to investigate replication efficiency, cell tropism, and associated immune responses of SARS-CoV-2 infection in cells of the CNS, we infected different types of human neural cultures. These cultures were differentiated from human induced pluripotent stem cells (hiPSCs) along a variety of different neural lineage specifications, which provided a unique and flexible platform to study the neurotropism of viruses *in vitro*. Specifically, we directed hiPSC colonies toward neural progenitor cells (NPCs) via an embryoid body stage and subsequently differentiated NPCs to mature neural networks (24). In addition, we also utilized a rapid neuronal differentiation protocol based on forced overexpression of the transcription factor Ngn2 in hiPSCs (Fig. 1A) (25, 26) to generate a pure population of neurons that we cocultured with hiPSC-derived astrocytes. Using these specified CNS cell types, we directly compared the characteristics of SARS-CoV-2 infection with the highly pathogenic H5N1 influenza A virus, a virus with zoonotic potential which is known to efficiently replicate in neural cells *in vivo* (27–32) and *in vitro* (33–36).

10.1128/mSphere.00270-21.2 FIG S2 Expression of important entry factors for SARS-CoV-2; original pictures from the gels. Expression of ACE2 with amplicon size of 124 bp (A), NRP1 with amplicon size of 187 bp (B), TMPRSS2 with amplicon size of 106 bp (C), and ACT with amplicon size of 153 bp (D) from one out of two independent experiments is shown. The important bands with the correct sizes of the amplicon were cropped and are represented in Fig. 1G. Download FIG S2, JPG file, 0.8 MB. Copyright © 2021 Bauer et al. 2021 Bauer et al. https://creativecommons.org/licenses/by/4.0/ This content is distributed under the terms of the Creative Commons Attribution 4.0 International license.

## RESULTS

### SARS-CoV-2 does not replicate efficiently in hiPSC-derived neural cell types, despite the presence of ACE2, TMPRSS2, and NRP1.

To investigate the replication efficiency of SARS-CoV-2, we utilized hiPSC-derived NPCs and differentiated these to mature forebrain cortical neural cultures using a previously published protocol (24) (Fig. 1A; see also Fig. S1 in the supplemental material). The resulting cultures contain a mix of electrically active neurons, astrocytes, and progenitors. Pure NPC and mixed mature neural network cultures were infected with SARS-CoV-2 and H5N1 virus at a multiplicity of infection (MOI) of 0.1 and 0.5. At 2, 6, 24, 48, and 72 h postinfection (hpi), infectious virus titers in the supernatants were determined by endpoint titration. In contrast to H5N1 virus, no productive infection in SARS-CoV-2-inoculated NPC and mature neural network cultures was detected (Fig. 1B and C). However, in one out of 3 experiments we observed an increase in virus titers at 72 h postinfection, which explains the small increase in virus titer in the mature neural network cultures. As an alternative to the laborious and time-consuming differentiation of mature neural networks through embryoid bodies and NPC stages, we also employed an established rapid differentiation protocol that yields a pure culture of iPSC-derived glutamatergic cortical neurons by overexpressing the transcription factor neurogenin-2 (Ngn2) (25). We further supplemented the Ngn2-induced neurons with hiPSC-derived astrocytes to support their survival and maturation. The final Ngn2 cocultures contain astrocytes and functional forebrain cortical neurons that form synapses (Fig. 1A and Fig. S1). SARS-CoV-2 did not replicate efficiently in the Ngn2 cocultures, in contrast to H5N1 virus (Fig. 1D). As a positive control for virus replication, VeroE6 and MDCK cells were infected with SARS-CoV-2 and H5N1 virus, respectively (Fig. 1E and F).

10.1128/mSphere.00270-21.1 FIG S1 Immunohistological characterization of neural cultures. (A) NPC cultures that were used to generate mixed neural cultures are positive for FOXG1 (green), indicating a forebrain identity. TUJ1, a marker for immature neurons, is hardly detected in cells, suggesting a pure population of precursor cells. Bar = 100 μm. (B) Eight-week-old mixed neural cultures contain astrocytes (GFAP, green) and neurons that stain positive for MAP2 (cyan) and the mature neuronal marker NEUN (red). Bar = 100 μm. (C) Neurons express pre- (synapsin, red) and postsynaptic (PSD95, green) proteins that colocalize with MAP2 (cyan), suggesting the formation of synapses. Bar = 10 μm. (D) Neurons (MAP2, cyan) in 3-week-old Ngn2 cocultures express FOXG1 (green) and NEUN (red), indicating that the cells have developed toward mature cortical forebrain neurons. Bar = 50 μm. (E) Ngn2 neurons express pre- (synapsin, red) and postsynaptic (PSD95, green) proteins that colocalize with MAP2 (cyan), suggesting the formation of synapses. Bar = 10 μm. Download FIG S1, JPG file, 2.9 MB. Copyright © 2021 Bauer et al. 2021 Bauer et al. https://creativecommons.org/licenses/by/4.0/ This content is distributed under the terms of the Creative Commons Attribution 4.0 International license.

Next, we evaluated the presence of important SARS-CoV-2 entry factors, such as angiotensin-converting enzyme 2 (ACE2), transmembrane protease serine 2 (TMPRSS2), and neuropilin-1 (NRP1). In all cultures, there was clear evidence for ACE2, TMPRSS2, and NRP1 expression, suggesting cellular susceptibility to SARS-CoV-2 infection (Fig. 1G and Fig. S2).

### SARS-CoV-2 infects MAP2-expressing neurons and does not induce cleavage of caspase-3.

**FIG 2:**
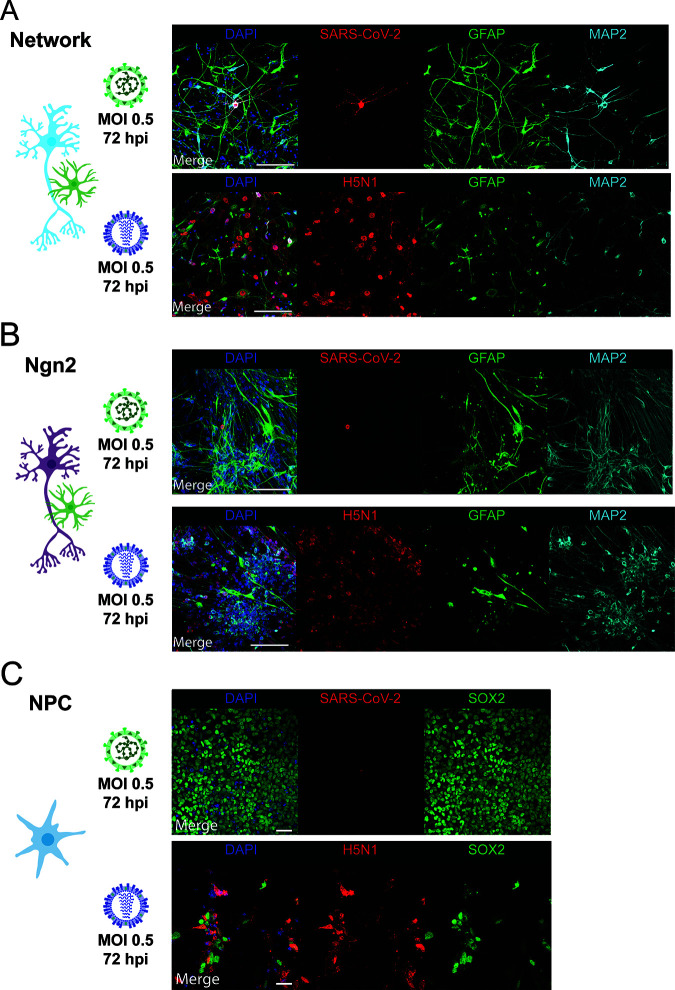
SARS-CoV-2 infects MAP2^+^ neurons. Mixed neural network cultures (bar = 100 μm) (A), Ngn2 cocultures (bar = 100 μm) (B), and NPCs (bar = 50 μm) (C) were infected at an MOI of 0.5 with SARS-CoV-2 or H5N1 virus, respectively. At 72 h postinfection, the cells were fixed and stained for the presence of viral antigen (SARS-CoV-2 NP or H5N1 NP in red). MAP2 (cyan) was used as a marker for neurons, astrocytes were identified by staining for glial fibrillary acidic protein (GFAP) (green), and SOX2 (green) was used as a marker for NPCs. Cells were counterstained with DAPI (blue) to visualize the nuclei. Data shown are representative examples from three independent experiments for each culture condition.

To determine whether SARS-CoV-2 was able to infect individual cells, we stained for virus nucleocapsid protein (NP) 24 and 72 hpi with an MOI of 0.5. SARS-CoV-2 sparsely infected cells in neural cultures at 24 and 72 hpi. Infection was observed in single scattered MAP2^+^ neurons (Fig. 2A and B and Fig. S3A and B). In one experiment, we identified a cluster of MAP2^+^ cells that stained positively for SARS-CoV-2 NP at 72 hpi (Fig. S4A). In the Ngn2 cocultures, we were able to detect only MAP2^+^/NEUN^+^ neurons positive for SARS-CoV-2 NP, suggesting that SARS-CoV-2 infects only mature neurons and does so only sparsely (Fig. S4B). We found no convincing evidence of SARS-CoV-2 NP^+^ cells among SOX2^+^ NPCs or glial fibrillary acidic protein-positive (GFAP^+^) astrocytes (Fig. 2A to C). In contrast, H5N1 virus abundantly infected SOX2^+^ NPCs, GFAP^+^ astrocytes, and MAP2^+^ neurons (Fig. 2A to C).

10.1128/mSphere.00270-21.3 FIG S3 SARS-CoV-2 infects MAP2^+^ neurons. Mixed neural culture (bar = 100 μm) (A) and Ngn2 cocultures (bar = 100 μm) (B) were infected at an MOI of 0.5 with SARS-CoV-2 or H5N1 virus, respectively. At 24 h postinfection, cells were fixed and stained for the presence of viral antigen (SARS-CoV-2 NP or H5N1 NP in red). MAP2 (cyan) was used as a marker for neurons, and astrocytes were identified by staining for glial fibrillary acidic protein (GFAP) (green). DAPI (blue) was used to visualize the nuclei. Data shown are representative examples from two independent experiments. Download FIG S3, JPG file, 2.8 MB. Copyright © 2021 Bauer et al. 2021 Bauer et al. https://creativecommons.org/licenses/by/4.0/ This content is distributed under the terms of the Creative Commons Attribution 4.0 International license.

10.1128/mSphere.00270-21.4 FIG S4 SARS-CoV-2 infects NEUN^+^ neurons and does not induce cleaved caspase-3 expression. (A) One focus of multiple infected cells was identified (bar = 100 μm). (B) The neurons in the Ngn2 cultures consist of a homogeneous population of mature NEUN^+^ neurons (bar = 50 μm). (C) High-magnification image of a SARS-CoV-2-infected cell in which no cleaved caspase-3 is visible (scale bar = 20 μm). Download FIG S4, PDF file, 10.2 MB. Copyright © 2021 Bauer et al. 2021 Bauer et al. https://creativecommons.org/licenses/by/4.0/ This content is distributed under the terms of the Creative Commons Attribution 4.0 International license.

**FIG 3:**
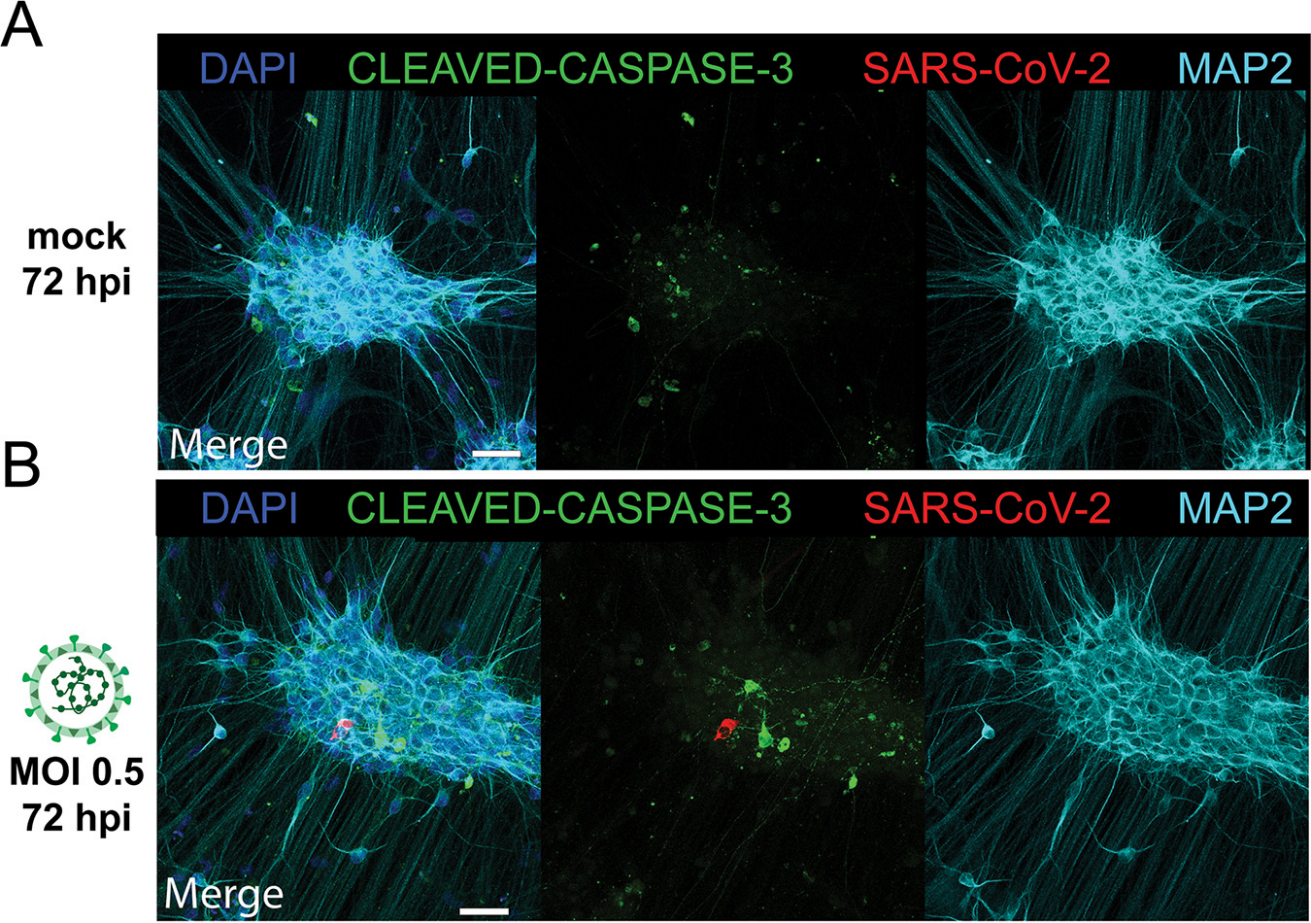
SARS-CoV-2 infections do not result in upregulation of cleaved caspase-3. Ngn2 cocultures were either mock infected (A) or infected with SARS-CoV-2 (B) at an MOI of 0.5 (bar = 50 μm). At 72 h postinfection, the cells were fixed and stained for the presence of SARS-CoV-2 antigen (red) and for the apoptosis marker cleaved caspase-3 (green). Data shown are representative examples from two independent experiments.

Next, despite the fact that there was no morphological evidence for cell death in the SARS-CoV-2-infected cultures, we wanted to investigate whether SARS-CoV-2 infection induced apoptosis in SARS-CoV-2 NP^+^ neurons. Therefore, we infected Ngn2 cocultures with SARS-CoV-2 and stained for cleaved caspase-3, an apoptosis marker. We again observed that SARS-CoV-2 infected only MAP2^+^ neurons. Neurons expressing SARS-CoV-2 NP did not show accumulation of cleaved caspase-3 (Fig. 3A and B and Fig. S4C).

### SARS-CoV-2 infection induces IFN-λ2/3 and IL-8.

**FIG 4:**
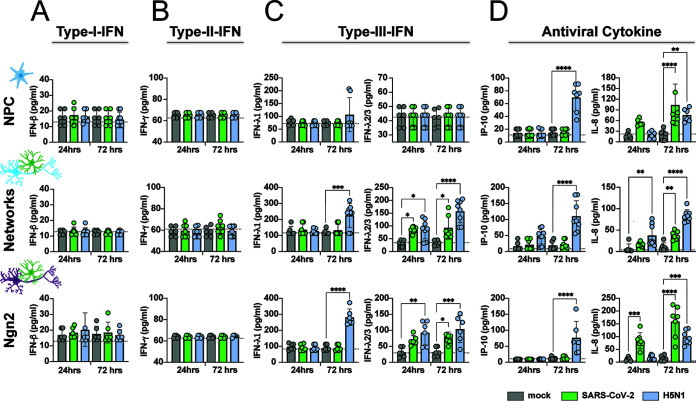
SARS-CoV-2 infection induces type III IFN and IL-8. NPCs, mixed neural network cultures, and Ngn2 cocultures were infected with SARS-CoV-2 and H5N1 virus at an MOI of 0.1. Concentrations of type I interferon (IFN-β) (A), type II IFN (IFN-γ) (B), type III IFN (IFN-λ1 and IFN-λ2/3) (C), and the antiviral cytokines IP-10 and IL-8 (D) were measured in the supernatant 24 and 72 h postinfection. The data are derived from three independent experiments, and each experiment was performed in either biological duplicates or triplicates. The assay was performed in technical duplicates for each sample. The data displayed represent average values from the technical duplicates of each experiment performed. Error bars denote mean ± standard deviation (SD). Statistical significance was calculated with a one-way analysis of variance (ANOVA) with a Bonferroni *post hoc* test, and the means from the mock-infected samples were compared to the means from the SARS-CoV-2- and H5N1 virus-infected samples at 24 and 72 h postinfection. Asterisks indicate statistical significance: *, *P* < 0.05; **, *P* < 0.01; ***, *P* < 0.001; ****, *P* < 0.0001.

To determine the immune response of the neural cultures toward SARS-CoV-2 and H5N1 virus infection, we measured a panel of antiviral cytokines in the supernatant of infected neural cultures at 24 and 72 h postinfection (Fig. 4). Even though SARS-CoV-2 infection was scarce, IFN-λ2/3 was induced in both the mixed neural culture and Ngn2 cocultures but not in NPC cultures. Increased secretion of interleukin-8 (IL-8) was observed in NPC cultures, mixed neural culture, and Ngn2 cocultures. H5N1 virus infection induced both type III IFN IFN-λ1 and IFN-λ2/3 in mixed neural cultures and Ngn2 cocultures, but not among NPCs. Furthermore, increased levels of IP-10 were detected only in the H5N1 virus-infected neural cultures. Similarly to SARS-CoV2, H5N1 virus was also able to induce IL-8 in all neural cultures. Neither SARS-CoV-2 nor H5N1 virus infection induced type I interferon (IFN-α/IFN-β) or type II IFN (IFN-γ) or other cytokines such as IL-1b, tumor necrosis factor alpha (TNF-α), IL-12p70, granulocyte-macrophage colony-stimulating factor (GM-CSF), or IL-10 (Fig. S5).

10.1128/mSphere.00270-21.5 FIG S5 SARS-CoV-2 infection as well as H5N1 virus infection results in modest immune responses. The concentrations of the cytokines which were measured in the supernatant of SARS-CoV-2- or H5N1 virus-infected NPCs (A), mixed neural cultures (B), and Ngn2 cocultures (C) are displayed. The values represent the means from three independent experiments. Download FIG S5, JPG file, 0.9 MB. Copyright © 2021 Bauer et al. 2021 Bauer et al. https://creativecommons.org/licenses/by/4.0/ This content is distributed under the terms of the Creative Commons Attribution 4.0 International license.

## DISCUSSION

SARS-CoV-2 replicated poorly in all three types of hiPSC-derived neural cultures used in our experiments, which contrasts largely with H5N1 virus, which replicated efficiently to high titers. Even though important entry factors for SARS-CoV-2 are expressed in all of the cultures used, SARS-CoV-2 infected a very small proportion of cells without evidence of subsequent spread. Additionally, we did not observe SARS-CoV-2-induced neuronal cell death, indicated by a lack of colabeling with apoptosis marker cleaved caspase 3 in SARS-CoV-2 NP^+^ neurons. However, SARS-CoV-2 infection did induce type III IFN and IL-8 production.

Evidence is accumulating that SARS-CoV-2 enters the CNS via the olfactory nerve (7–9), a pathway that is also used by influenza A viruses to enter the CNS in many mammals including humans (10, 37). H5N1 virus spreads efficiently to the CNS via the olfactory nerve in experimentally inoculated ferrets and subsequently replicates very efficiently in the CNS (27, 29, 38). Unlike H5N1 virus infection, SARS-CoV-2 is rarely detected in the CNS of fatal COVID-19 patients or experimentally inoculated animals (11–15). In addition, only a few case reports of SARS-CoV-2-induced encephalitis have been reported (39, 40). *In vitro* studies have mainly focused on the ability of SARS-CoV-2 to infect cells of the CNS based on the detection of viral RNA or viral antigen (18, 19, 22, 23, 41), but not many have investigated replication efficiency in time by measuring infectious virus titers (17). So far, several studies showed limited infection of neurons in hiPSC-derived brain organoids (22) and BrainSpheres (19), without efficient SARS-CoV-2 replication. So far, only efficient replication has been shown in choroid plexus organoids, especially within choroid plexus epithelium cells (17, 23). Altogether, these observations are consistent with our findings of poor SARS-CoV-2 replication in hiPSC-derived NPCs, neurons, and astrocytes and support a pathophysiological model whereby SARS-CoV-2 invades the CNS but does not replicate efficiently in CNS cell types. However, one caveat of our study is that other cells such as microglia, oligodendrocytes, and vascular cells (pericytes and endothelial cells) are not present. Therefore, we cannot exclude that SARS-CoV-2 can infect and possibly replicate efficiently in other cells of the CNS or neuronal cell types such as cortical parvalbumin (PV) interneurons or midbrain or hindbrain cell types.

Despite the low proportion of SARS-CoV-2-infected cells and the fact that infection seemed to be abortive in the hiPSC-derived neural cultures, we found evidence for cellular immune activation. In particular, SARS-CoV-2 infection of the neural cultures resulted in the induction of type III IFN, especially IFN-λ2/3, but not type I IFN or type II IFN. This result is in accordance with earlier reports suggesting that SARS-CoV-2 triggers only very mild type I and type II IFN responses but does trigger a robust type III IFN response in cell culture, human airway epithelial cells, ferrets, and SARS-CoV-2-infected individuals (42, 43). In addition, IL-8—a chemotactic factor that attracts leukocytes—was induced in all hiPSC-derived cultures. In lung tissue and peripheral venous blood serum of SARS-CoV-2-infected patients, elevated levels of IL-8 are associated with severe COVID-19 (44–46). Furthermore, IL-8 has been detected in the CSF of SARS-CoV-2 patients who developed encephalitis, which might be induced by the SARS-CoV-2-associated brain immune response, since SARS-CoV-2 RNA could not be detected in the CSF (47). However, how exactly these cytokines contribute to the *in vivo* neuroinflammatory process and if they are directly triggered by SARS-CoV-2 entry into the CNS need further investigations.

Highly pathogenic H5N1 virus replication has been reported *in vivo* (27, 29, 31, 32, 48) and *in vitro* across several different types of human and mouse neural cell cultures (34–36), including the human neuroblastoma line SK-N-SH (26), suggesting this virus is neurotropic. This fits with our observation that H5N1 virus replicates productively and spreads throughout hiPSC-derived neural cultures, infecting NPCs as well as mature neurons and astrocytes. H5N1 virus infection also results in the upregulation of the type III IFN IFN-λ1 and IFN-λ2/3, as well as the antiviral cytokines IL-8 and IP-10. IP-10 has been detected in the CSF of influenza A virus-infected patients and was found to be elevated in the brains of mice experimentally infected with H5N1 virus (49). However, the mechanism by which H5N1 virus achieves abundant virus replication and robust induction of proneuroinflammatory cytokines remains poorly understood.

Overall, our data fit with clinical findings concerning the ability of these viruses to cause acute CNS disease. In both humans and mammalian animal models, H5N1 virus is able to invade and replicate in cells of the CNS, including neurons, resulting in an acute (meningo-)encephalitis (50–52). In contrast, even though evidence exists that SARS-CoV-2 is able to spread to the CNS via the olfactory nerve (7–9) in SARS-CoV-2-infected patients or experimentally infected animal models, virus is rarely detected in the CNS or associated with acute encephalopathies (13–15).

Altogether, our findings reveal that replication of SARS-CoV-2 in CNS cell types is very limited, which is in contrast to the efficient replication and spread of H5N1 virus. Although the mechanistic pathogenesis of SARS-CoV-2-associated CNS disease remains poorly understood, this study supports the hypothesis that SARS-CoV-2 entry into the CNS and direct infection of a small subset of neurons might trigger inflammation in the brain.

## MATERIALS AND METHODS

### Cell lines.

VeroE6 (ATCC CRL 1586) cells were maintained in Dulbecco’s modified Eagle’s medium (DMEM; Lonza, Breda, the Netherlands) supplemented with 10% fetal calf serum (FCS; Sigma-Aldrich, St. Louis, MO, USA), 10 mM HEPES, 1.5 mg/ml sodium bicarbonate, 100 IU/ml penicillin (Lonza, Basel, Switzerland), and 100 μg/ml streptomycin (Lonza). Madin-Darby canine kidney (MDCK) cells were maintained in Eagle minimal essential medium (EMEM; Lonza) supplemented with 10% FCS, 100 IU/ml penicillin, 100 μg/ml streptomycin, 2 mM glutamine, 1.5 mg/ml sodium bicarbonate (1 mM), 10 mM HEPES, and 0.1 mM nonessential amino acids. All cell lines were grown at 37°C in 5% CO_2_. The medium was refreshed every 3 to 4 days, and cells were passaged at >90% confluence with the use of phosphate-buffered saline (PBS) and trypsin-EDTA (0.05%). The cells were routinely checked for the presence of mycoplasma.

### Differentiation of iPSCs to NPCs and mature neural cultures.

Human induced pluripotent stem cells (iPSCs) [WTC-11 Coriell no. GM25256, obtained from the Gladstone Institute, San Francisco, CA, USA] were differentiated to NPCs as previously described (2) with slight modifications. After passage 3, NPC cultures were purified using fluorescence-activated cell sorting (FACS) as described previously (53). Briefly, NPCs were detached from the culture plate and resuspended into a single-cell solution. CD184^+^/CD44^−^/CD271^−^/CD24^+^ cells were collected using a FACSAria III (BD Bioscience) and expanded in NPC medium (Table 1). NPCs were used for experiments between passages 3 and 7 after sorting or differentiated to neural networks. For differentiation toward mature neural cultures, NPCs were grown in neural differentiation medium (Table 1) for 6 to 8 weeks to achieve mature neural networks (24) and subsequently used for experiments; after week 4, only half of the medium was refreshed. Cultures were kept at 37°C and 5% CO_2_ throughout the differentiation process.

**TABLE 1:** Overview of media and reagents used

Name	Reagent^*a*^	Manufacturer, catalogue no.
NPC medium	Advanced DMEM/F-12	ThermoFisher Scientific, 1634010
	1% N-2 supplement	ThermoFisher Scientific, 17502048
	2% B-27 minus RA supplement	ThermoFisher Scientific, 12587010
	1 μg/ml laminin	Sigma-Aldrich, L2020
	1% penicillin-streptomycin	ThermoFisher Scientific, 15140122
	20 ng/ml basic fibroblast growth factor	Merck, GF003AF

Neural differentiation medium	Advanced DMEM/F-12	ThermoFisher Scientific, 1634010
	1% N-2 supplement	ThermoFisher Scientific, 17502048
	2% B-27 minus RA supplement	ThermoFisher Scientific, 12587010
	2 μg/ml laminin	Sigma-Aldrich, L2020
	1% penicillin-streptomycin	ThermoFisher Scientific, 15140122
	10 ng/ml BDNF	ProSpec Bio, CYT-207
	10 ng/ml GDNF	ProSpec Bio CYT-305
	1 μM db-cAMP	Sigma, D0627
	200 μM ascorbic acid	Sigma, A5960

Ngn2 medium	Neurobasal medium	ThermoFisher Scientific, 21103049
	2% B-27 minus RA supplement	ThermoFisher Scientific, 12587012
	1% GlutaMAX	ThermoFisher Scientific, 35050061
	10 ng/ml NT3	PeproTech, 450-03
	10 ng/ml BDNF	ProSpec, CYT-207
	1% penicillin-streptomycin	ThermoFisher Scientific, 15140122

a BDNF, brain-derived neurotrophic factor; GDNF, glial-derived neurotrophic factor.

### Differentiation of iPSCs to Ngn2 cocultures.

iPSCs were directly differentiated into excitatory cortical layer 2/3 neurons by forcibly overexpressing the neuronal determinant neurogenin-2 (Ngn2) (25, 26). To support neuronal maturation, hiPSC-derived astrocytes were added to the culture in a 1:1 ratio. At day 3, the medium was changed to Ngn2 medium (Table 1). Cytosine β-d-arabinofuranoside (Ara-C) (2 μM; Sigma; C1768) was added once to remove proliferating cells from the culture and ensure long-term recordings of the cultures. From day 6 onward, half of the medium was refreshed three times per week. Cultures were kept at 37°C and 5% CO_2_ throughout the differentiation process.

### Viruses.

The SARS-CoV-2 isolate (isolate BetaCoV/Munich/BavPat1/2020; European Virus Archive Global no. 026V-03883; kindly provided by C. Drosten) was previously described by Lamers et al. (54, 55) The zoonotic highly pathogenic avian influenza (HPAI) H5N1 virus (A/Indonesia/5/2005) was isolated from a human patient, and the virus was propagated once in embryonated chicken eggs and twice in MDCK cells.

### Virus titrations.

The SARS-CoV-2 titers were determined by endpoint dilution on VeroE6 cells, calculated according to the method of Kärber (57) and expressed as 50% tissue culture infectious doses/milliliter (TCID_50_/ml). SARS-CoV-2 virus titers were determined by preparing 10-fold serial dilutions in triplicates of supernatants in Opti-MEM containing GlutaMAX. Dilution supernatants were added to a monolayer of 40,000 VeroE6 cells/well in a 96-well plate and incubated at 37°C. After 5 days, the plates were examined for the presence of cytopathic effect (CPE). Virus titers of HPAI H5N1 were determined by endpoint dilution on MDCK cells as described previously (56). In short, 10-fold serial dilutions of cell supernatant in triplicates were prepared in influenza infection medium which consists of EMEM supplemented with 100 IU/ml penicillin, 100 μg/ml streptomycin, 2 mM glutamine, 1.5 mg/ml sodium bicarbonate, 10 mM HEPES, 1× (0.1 mM) nonessential amino acids, and 1 μg/μl tosylsulfonyl phenylalanyl chloromethyl ketone (TPCK)-treated trypsin (Sigma-Aldrich). Prior to adding the virus dilutions to the MDCK cells, the cells were washed once with plain EMEM to remove residual FCS. One hundred microliters of the diluted supernatants was used to inoculate 30,000 MDCK cells/well in a 96-well plate. After 1 h, the inoculum was removed and 200 μl fresh influenza infection medium was added. Four days after infection, supernatants of the infected MDCK cells were tested for agglutination. Twenty-five microliters of the supernatant was mixed with 75 μl 0.33% turkey red blood cells and incubated for 1 h at 4°C. The titers of infectious virus were calculated according to the method of Kärber (57) and expressed as TCID_50_/ml. All experiment with infectious SARS-CoV-2 and H5N1 virus were performed in a class II biosafety cabinet under biosafety level 3 (BSL-3) conditions at the Erasmus Medical Center. The initial 1:10 dilution of cell supernatant resulted in a detection limit of 10^1.5^ TCID_50_/ml.

### Replication kinetic.

Before infection of neural progenitors, network neurons, and Ngn2 neurons, supernatant was removed and cells were infected with SARS-CoV-2 and H5N1 virus at the indicated multiplicity of infection (MOI). As a control for active virus replication, VeroE6 and MDCK cells were infected with SARS-CoV-2 and H5N1 virus, respectively. Before virus infection, the VeroE6 cells were washed with SARS-CoV-2 infection medium (DMEM supplemented with 2% FBS and 100 IU/ml penicillin, 100 μg/ml streptomycin) and MDCK cells were washed with influenza infection medium. After 1 h of incubation at 37°C, the inoculum was removed and replaced with fresh medium and old medium in a 1:1 ratio. After removing the inoculum from VeroE6 and MDCK cells, SARS-CoV-2 infection medium and influenza infection medium, respectively, were added to the cells. At the indicated time points, an aliquot of the supernatant was collected for subsequent virus titration. All experiments were performed in biological triplicates and in either technical duplicates or triplicates.

### PCR validation of ACE2, TMPRSS2, and NRP1 expression.

RNA was isolated from the neural cultures and VeroE6 cells using the High Pure RNA isolation kit (Roche). The concentration of RNA was determined using a NanoDrop spectrophotometer. A 2.5-μg amount of RNA was reverse transcribed into cDNA using the SuperScript III reverse transcriptase (Invitrogen) according to the manufacturer’s protocol. cDNA of bronchus-bronchiole organoids was kindly provided by Anna Z. Mykytyn. The presence of ACE2, TMPRSS2, NRP1, and ACT was evaluated by amplifying these genes with gene-specific primers (Table 2) by PCR. Gene products were visualized on a 2% agarose gel which was stained with SYBR Safe. PCR products of the genes were sequenced to validate that the right product was amplified.

**TABLE 2:** Gene-specific primers for PCR

Species	Gene	Sequence (5′→3′)	Annealing temp (°C)	Amplicon (bp)	Reference
Human	ACE2-FWD	GGGATCAGAGATCGGAAGAAGAAA	60	124	1
Human	ACE2-REV	AGGAGGTCTGAACATCATCAGTG			1
Human	b-ACTIN-FWD	CCCTGGACTTCGAGCAAGAG	60	153	1
Human	b-ACTIN-REV	ACTCCATGCCCAGGAAGGAA			1
Human	TMPRSS2-FWD	AATCGGTGTGTTCGCCTCTAC	60	106	1
Human	TMPRSS2-REV	CGTAGTTCTCGTTCCAGTCGT			1
Human	NRP1-FWD	GACTGGGGCTCAGAATGGAG	60	187	
Human	NRP1-REV	ATGACCGTGGGCTTTTCTGT			

### Multiplexed bead assay for cytokine profiling.

Cytokines were measured using the LEGENDplex human antivirus response panel (BioLegend). The kit was used according to the manufacturer’s manual with an additional fixing step. After adding the SA-PE and performing the washing steps, the supernatant and the beads were fixed with formalin for 15 min at room temperature and washed twice with the provided wash buffer. This ensures that all pathogens are not infectious.

### Immunofluorescent labeling.

Cells were fixed using 4% formalin in PBS and labeled using immunocytochemistry. Primary antibody incubation was performed overnight at 4°C. Secondary antibody incubation was performed for 2 h at room temperature. Both primary and secondary antibody incubations were performed in staining buffer (0.05 M Tris, 0.9% NaCl, 0.25% gelatin, and 0.5% Triton X-100 [Sigma; T8787] in PBS [pH 7.4]). Primary antibodies and their dilutions can be found in Table 3. Secondary antibodies conjugated to Alexa-488, Alexa-647, or Cy3 were used at a dilution of 1:400 (Jackson ImmunoResearch). Nuclei were visualized using 4′,6-diamidino-2-phenylindole (DAPI) (ThermoFisher Scientific; D1306). Samples were embedded in Mowiol 4-88 (Sigma-Aldrich; 81381) and imaged using a Zeiss LSM 800 confocal microscope (Oberkochen, Germany).

**TABLE 3:** Antibodies

Antibody	Dilution	Manufacturer, catalogue no.
SARS-CoV-2 anti-NP	1:500	Sino Biological, 40143-MM05
SOX2	1:250	Millipore, AB5603
NEUN	1:100	Merck, ABN78
GFAP	1:200	Millipore, AB5804
MAP2	1:200	Synaptic Systems, 188004
H5N1 anti-NP	1:1,000	EVL, EBS-I-047, clone Hb65
Cleaved caspase-3	1:100	Cell Signaling Technologies, 9661S
Beta III tubulin (TUJ1)	1:200	Millipore, AB9354
PSD-95	1:100	Thermo Scientific, MA1-046
Synapsin	1:100	Synaptic Systems, 106 103
FOXG1	1:200	Abcam, 18259
