# Supplementary figures and images for: Replication Kinetics, Cell Tropism, and Associated Immune Responses in SARS-CoV-2- and H5N1 Virus-Infected Human Induced Pluripotent Stem Cell-Derived Neural Models
Source: mSphere. 2021 Jun 23;6(3):e00270-21. doi: 10.1128/mSphere.00270-21 (PMC8265642; doi:10.1128/mSphere.00270-21)

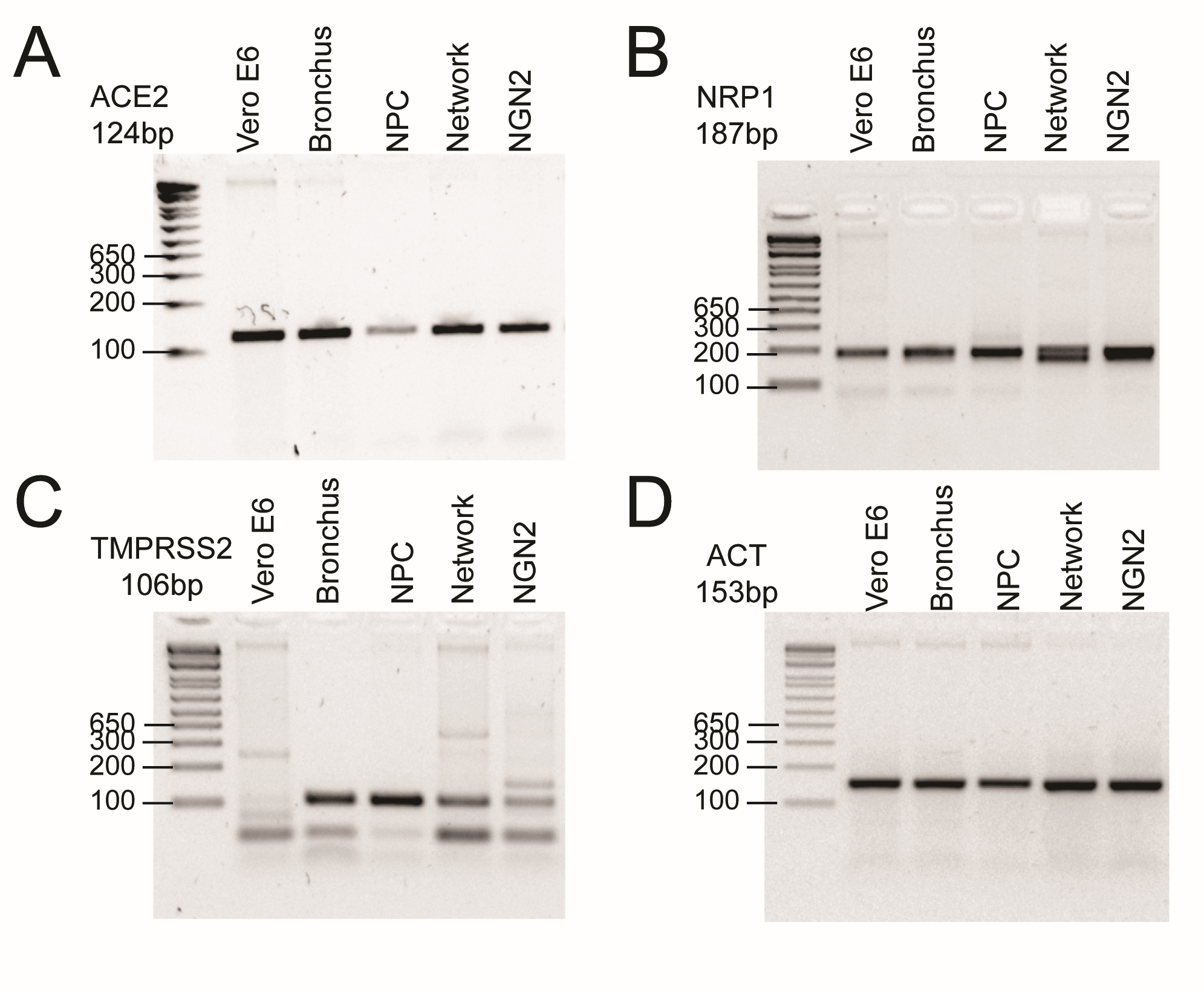

Supplement: FIG S2 [file msphere.00270-21-sf002.jpg]

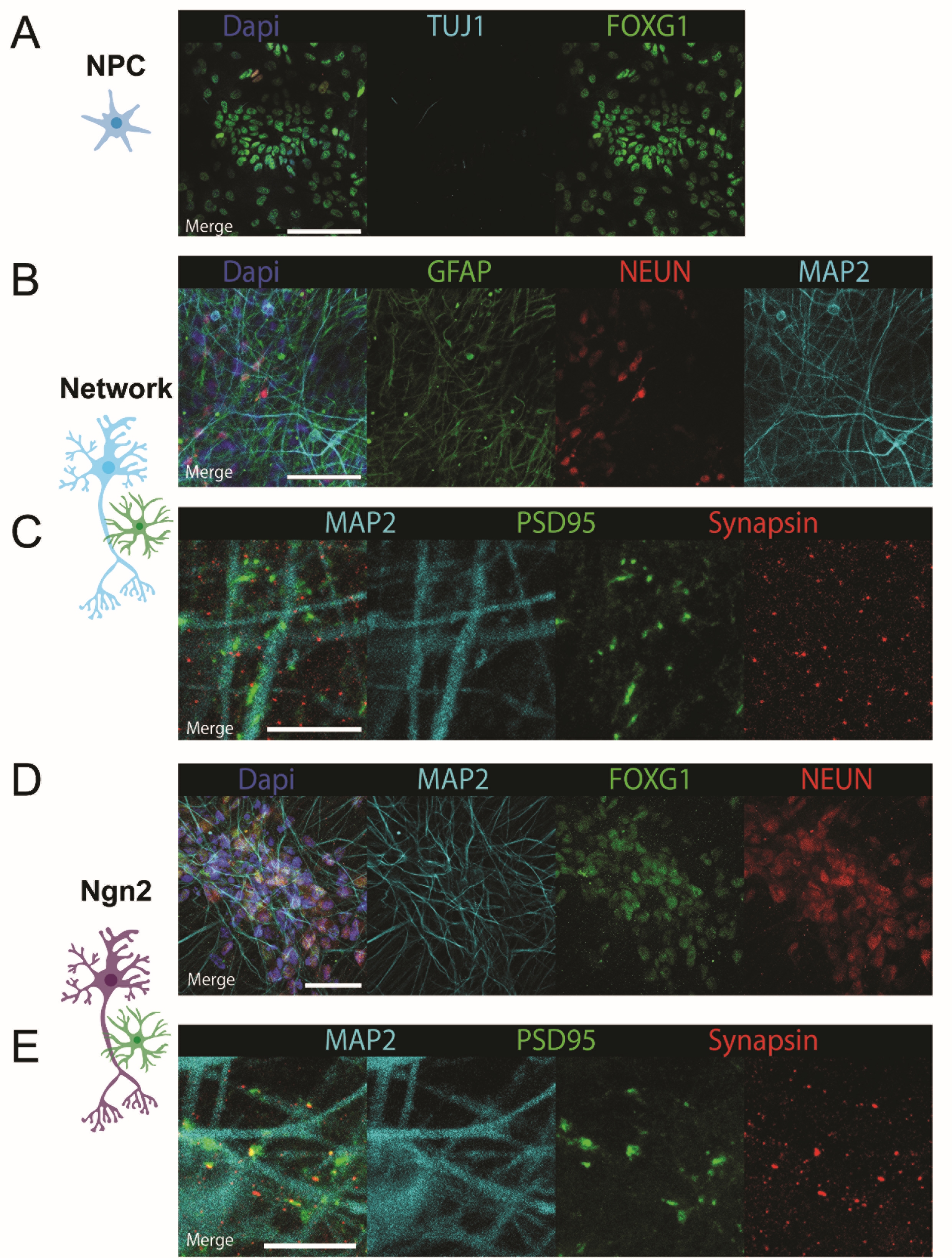

Supplement: FIG S1 [file msphere.00270-21-sf001.jpg]

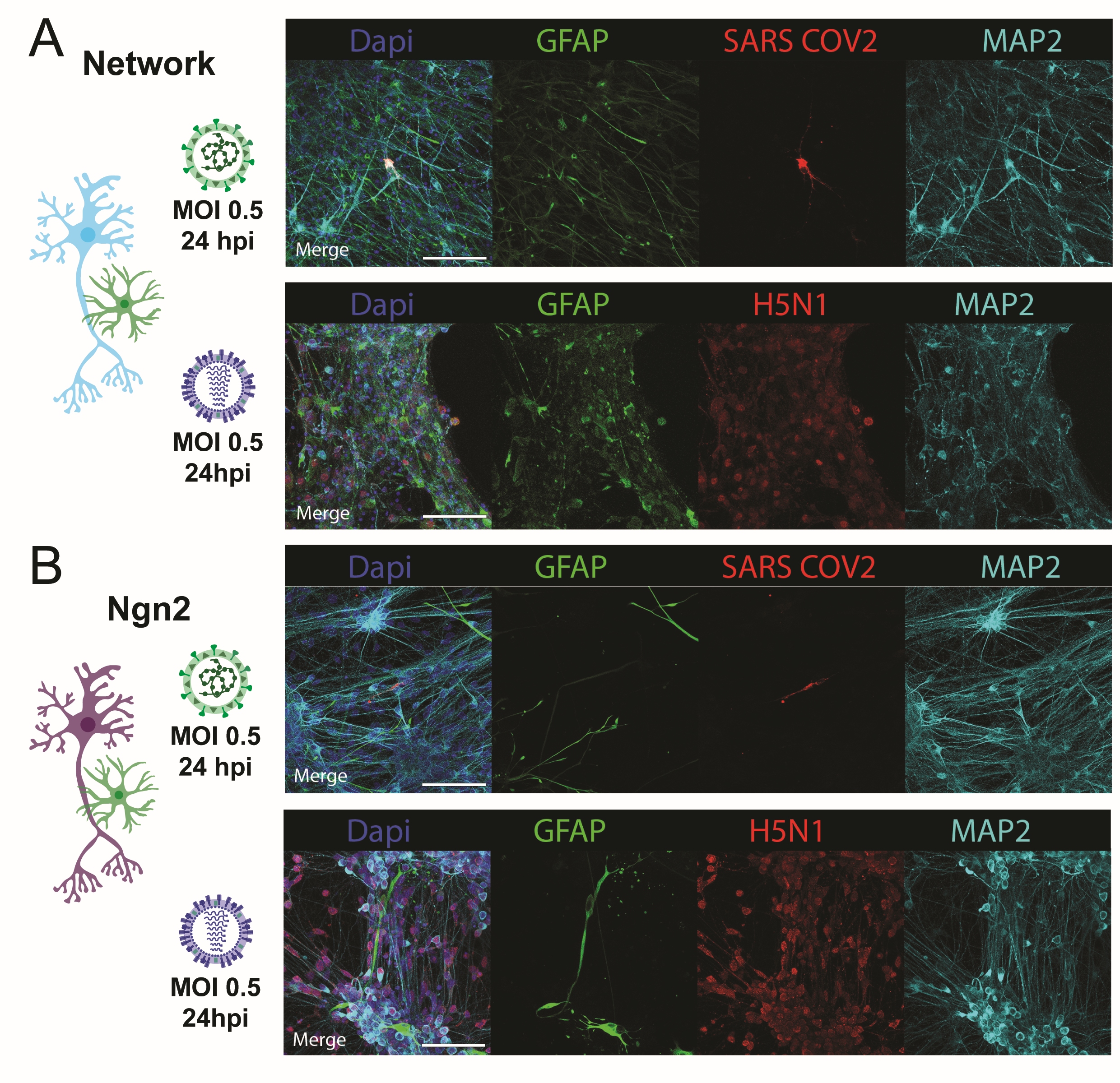

Supplement: FIG S3 [file msphere.00270-21-sf003.jpg]

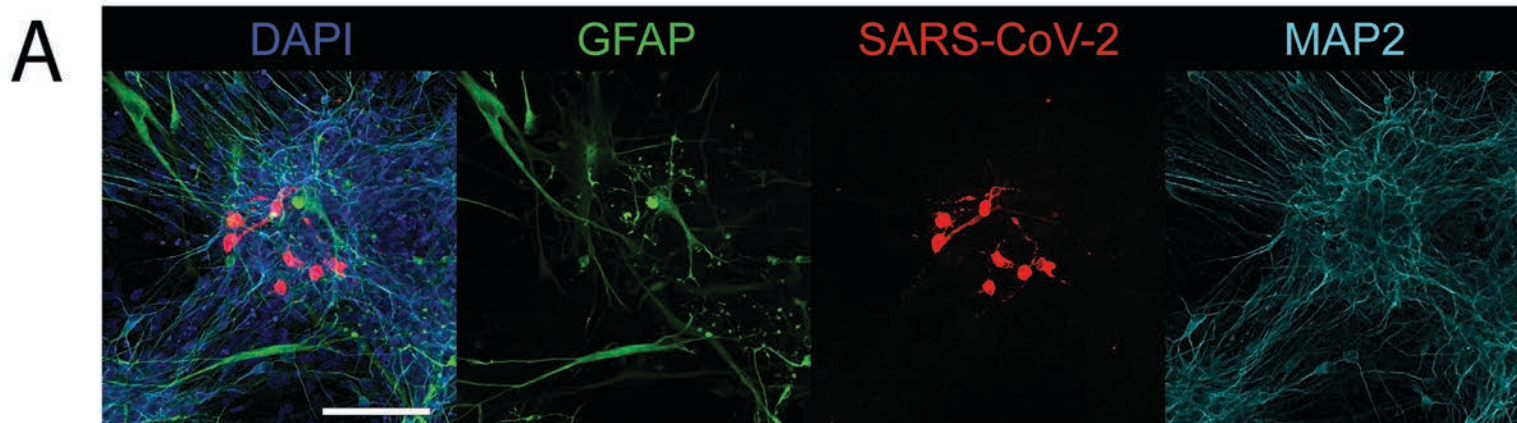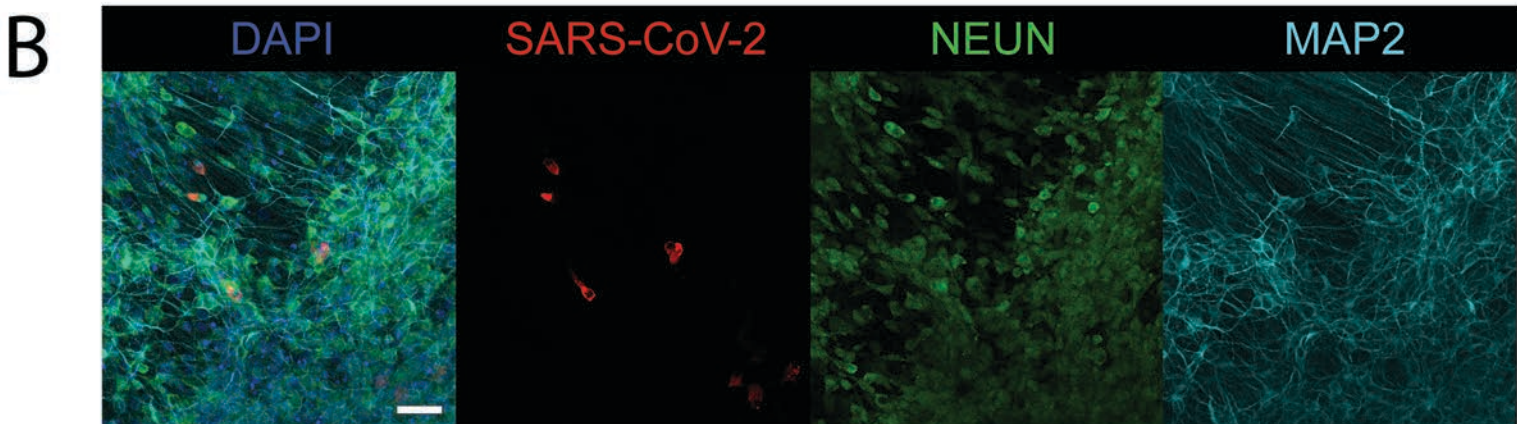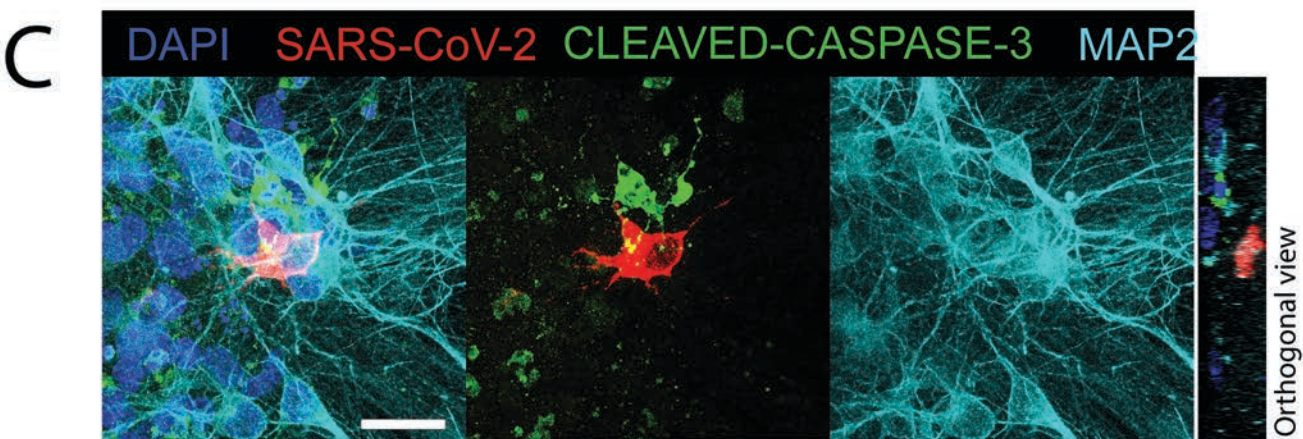

Supplement: FIG S4 [file msphere.00270-21-sf004.pdf]

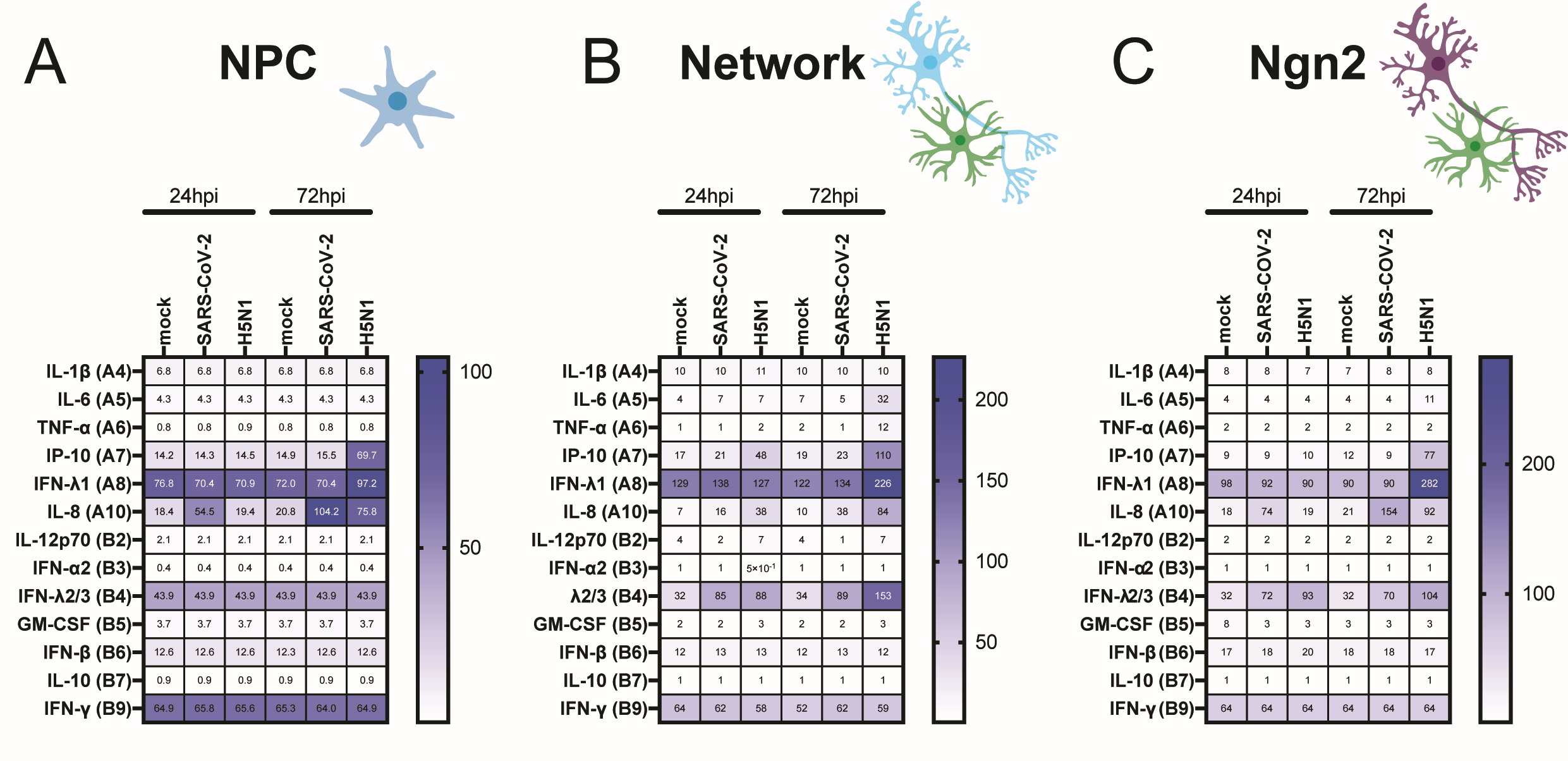

Supplement: FIG S5 [file msphere.00270-21-sf005.jpg]
